# Solitary fibrous tumour of the pleura arising in a pulmonary cavity

**DOI:** 10.1002/rcr2.635

**Published:** 2020-08-03

**Authors:** Hiroyuki Miura, Jun Miura, Keisei Tachibana, Shinichi Goto

**Affiliations:** ^1^ Department of Thoracic Surgery Akiru Municipal Medical Center Akiruno Japan; ^2^ Department of Surgery Kyorin University School of Medicine Mitaka Japan; ^3^ Department of Respirology Akiru Municipal Medical Center Akiruno Japan

**Keywords:** Cavity formation, pulmonary cyst, solitary fibrous tumour of the pleura, thoracic surgery

## Abstract

Solitary fibrous tumour of the pleura (SFTP) arising in a cavity is extremely rare. A 66‐year‐old Japanese male presented with an abnormal shadow on his chest X‐ray. Chest computed tomography showed a cavity of approximately 18 mm in diameter between the upper and lower lobes that contained a solid nodule within. Under the thoracoscope, the peduncle cystic tumour was removed with sufficient surgical margin. Macroscopically, a tumour of about 15 mm in diameter arose in the cystic cavity. Immunohistochemical stains were positive for CD34, bcl‐2, and signal transduction and activator of transcription 6 (STAT6) but negative for smooth muscle actin (SMA), desmin, and epithelial membrane antigen (EMA), and a diagnosis of SFTP was made. The patient remains well without recurrence or any complications at two and a half years after the operation. SFTP should be considered when a tumour arises in a cavity existing in an interlobar space. It is important to determine whether the tumour is pedunculated or sessile during surgery and to perform the appropriate surgical procedure.

## Graphical abstract

Solitary fibrous tumour of the pleura (SFTP) is a rare neoplasm. Moreover, cavity formation associated with SFTP is extremely rare. To our knowledge, only one such case has been previously reported.

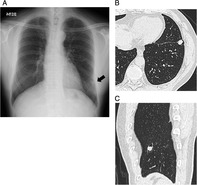

## Introduction

Solitary fibrous tumour of the pleura (SFTP) is a rare neoplasm that accounts for less than 5% of primary pleural tumours and has an annual incidence of 2.8 cases per 100,000 individuals [1]. Cavity formation associated with SFTP is extremely rare. To our knowledge, only one such case has been previously reported.

## Case Report

**Figure 1:**
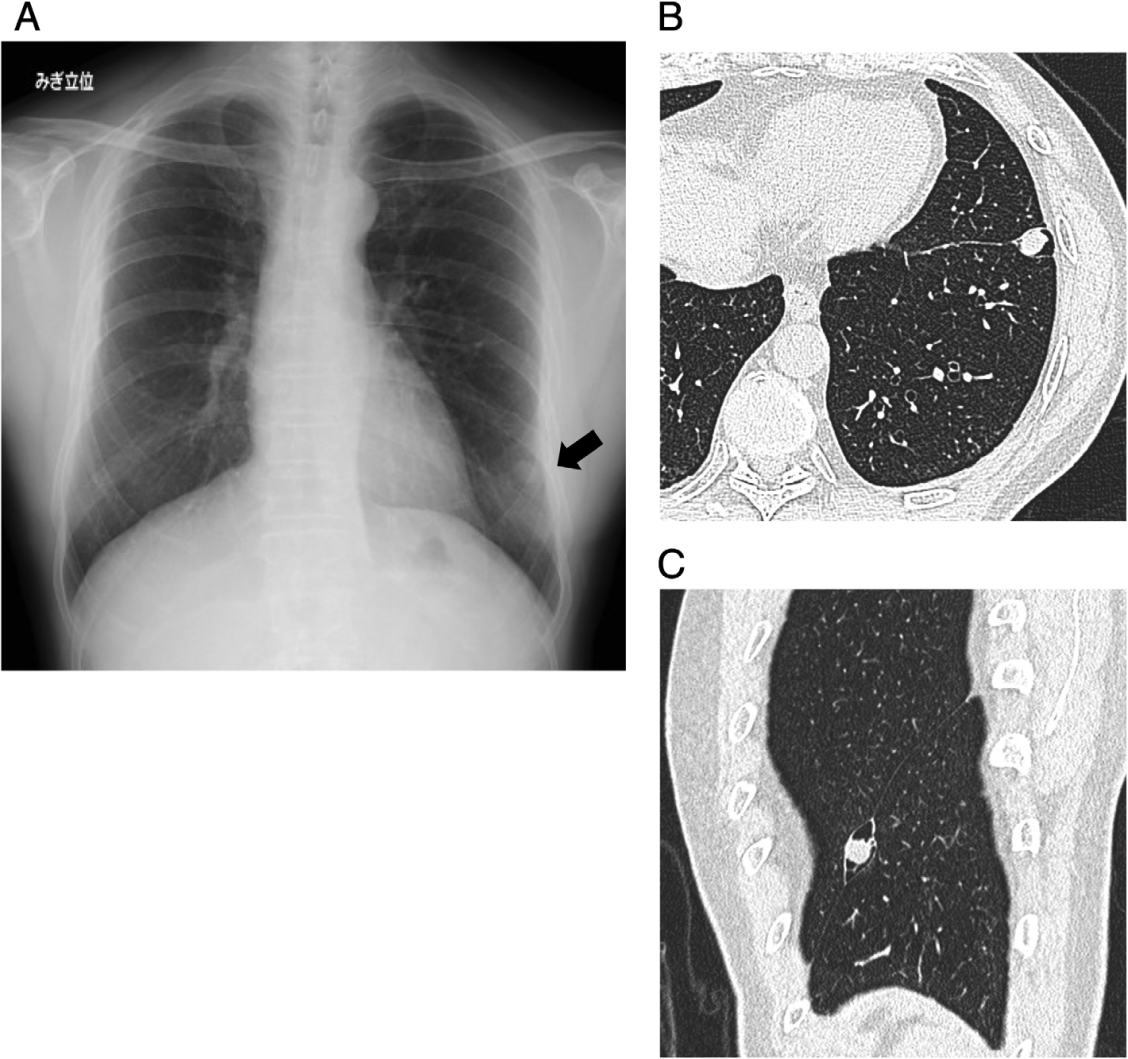
(A) The chest X‐ray showed a well‐defined 15 mm × 11 mm nodule in the left lower lung field. (B, C) Computed tomography (CT) shows a tumour arising in a cavity located in the interlobar space.

A 66‐year‐old non‐smoking Japanese male presented with an abnormality found on a chest X‐ray performed during an annual check‐up. The patient had no past medical history and an unremarkable family history. His chest X‐ray showed a well‐defined 15 mm × 11 mm nodule in the left lower lung field (Fig. 1A). Chest computed tomography (CT) showed a cavity of approximately 18 mm in diameter between the upper and lower lobes that contained a solid nodule within (Fig. 1B, C). The tumour is enhanced in mosaic on mediastinal window. Blood testing, including tumour markers [carcinoembryonic antigen (CEA), Cyfla, and pro‐gastrin releasing peptide (proGRP)], haemogram, and renal and hepatic function enzymes were within normal range. The maximum standard uptake value (SUVmax) of the nodule on fluorodeoxyglucose (FDG) positron emission tomography was 1.22, consistent with benign tumour.

**Figure 2:**
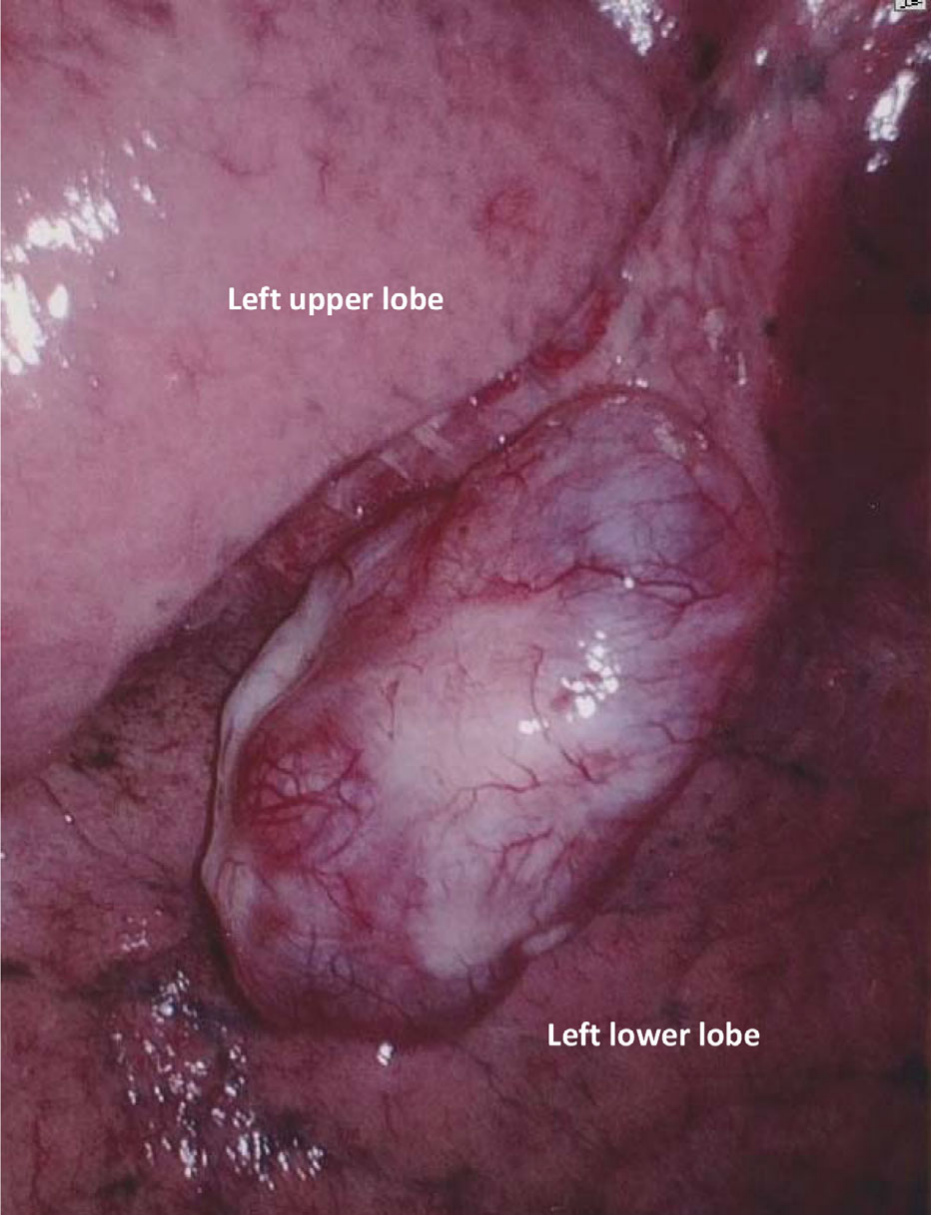
A pedunculated tumour was observed in the interlobar space.

Under the thoracoscope, the pedunculated cystic tumour arose from the S4 area and was removed with sufficient surgical margins. Macroscopically, the tumour arose in the cystic cavity and was approximately 15 mm in diameter (Fig. 2). Microscopically, it was composed of scanty spindle cells without atypia. Immunohistochemical stains were positive for CD34, bcl‐2, and signal transduction and activator of transcription 6 (STAT6), but negative for smooth muscle actin (SMA), desmin, and epithelial membrane antigen (EMA). Both the cells lining the inside of cyst and those lining the clefts seen within the tumour exhibited staining by cytokeratin 7 (CK7) and thyroid transcription factor‐1 (TTF‐1). The final diagnosis was SFTP. The cyst wall was also composed of tumours. Furthermore, small bullae were found around the resected tumour. The patient remains well without recurrence or complication at two and a half years after the operation.

## Discussion

Baek et al. reported a case similar to the one reported here in a 58‐year‐old non‐smoking woman with an air‐containing SFTP located in the right major fissure [2]. They speculated that entrapment of peripheral lung tissue within the tumour was the cause of cyst formation, as the epithelial cells lining the cyst stained positive for TTF‐1 and CK7, consistent with pneumocytes originating from alveoli or terminal respiratory epithelium. The epithelial lining of the cyst in our case also stained positive for TTF‐1 and CK7, as did the epithelium lining the small clefts in the tumour. Thus, entrapment of peripheral lung tissue cannot be denied. However, another possibility is that the tumour progressed in the mesenchymal area of a previously existing bulla and a part of the tumour bulged to form an intracystic tumour. Actually, there were small bullae around the resected tumour. The check‐valve mechanism that Baek et al. proposed requires communicating bronchioles. However, the pedunculated tumour in this case was located at the lung surface, where there are no bronchioles.

Although most of the SFTP are benign, up to 10% are malignant [1]. de Perrot et al. define malignancy as high cellularity with crowding and overlapping of nuclei, cellular pleomorphism, high mitotic count (>4 per 10 high‐power fields), necrosis, or stromal/vascular invasion. According to the World Health Organization (WHO) classification, >4 mitoses per 2 mm^2^ is the most reliable indicator of aggressive behaviour [1]. Complete surgical resection is the mainstay of treatment for both benign and malignant SFTP and 1–2 cm surgical margins are recommended. Pedunculated tumours are easily resected with wedge resections of the lung. However, sessile or inverted tumours require more extended resection with segmentectomy, lobectomy, or pneumonectomy. For tumours arising from the parietal pleura, chest wall resection with adequate surgical margin should be considered.

Mahesh et al. reported a case of recurrence in a benign SFTP more than 10 years after resection [3]. Moreover, malignant transformation was noted during histopathological examination of specimens from the third and fourth resections. Therefore, surgeons should not hesitate to perform extensive resection of the chest wall adherent to the tumour.

de Perrot et al. proposed a staging system and algorithm for management and follow‐up of SFTP in 2002 [4]. The staging system is as follows: stage 0, pedunculated tumour without signs of malignancy; stage I, sessile or inverted tumour without signs of malignancy; stage II, pedunculated tumour with histological signs of malignancy; stage III, sessile or inverted tumour with histological signs of malignancy; and stage IV, multiple synchronous metastatic tumours. They also found that the risk of recurrence for benign pedunculated, benign sessile, and malignant pedunculated SFTP is <2%, <8%, and 14%, respectively, and suggested that no adjuvant therapy is required for these three categories. However, they did suggest that adjuvant therapy should be considered for malignant sessile SFTP because of a 63% recurrence risk [4]. Nonetheless, adjuvant therapy has not been established due to the rarity of this tumour. According to the above‐mentioned criteria, our case would be considered stage 0 with a <2% risk of recurrence and no adjuvant therapy is needed. Only an annual radiographic follow‐up is recommended [4].

Tapias et al. proposed a scoring system to predict recurrence after SFTP resection in 2013 [5]. They constructed a predictive score by assigning one point to each of the following six variables: parietal (vs. visceral) pleural origin; sessile (vs. pedunculated) morphology; size >10 cm (vs. <10 cm); and presence of hypercellularity, necrosis, and mitotic activity ≥4/high‐power field (vs. <4). With a score < 3, recurrence‐free survival was 100% up to 15 years. According to this scoring system, our case scored 0 and a favourable prognosis is expected.

SFTP, although rare, should be considered in cases of a tumour arising in a cavity existing in an interlobar space, especially with a gradually thickening cyst wall. It is important to determine whether the tumour is pedunculated or sessile during surgery and to perform the appropriate surgical procedure.

### Disclosure Statement

Appropriate written informed consent was obtained for publication of this case report and accompanying images.
